# Altered hemispheric asymmetries as an endophenotype in psychological and developmental disorders: A theory on the influence of stress on brain lateralization

**DOI:** 10.3389/fnbeh.2022.1054114

**Published:** 2022-11-04

**Authors:** Gesa Berretz, Julian Packheiser

**Affiliations:** ^1^Department of Biopsychology, Institute of Cognitive Neuroscience, Faculty of Psychology, Ruhr University Bochum, Bochum, Germany; ^2^Social Brain Lab, Netherlands Institute for Neuroscience, Amsterdam, Netherlands

**Keywords:** asymmetry, mental disorders, depression, schizophrenia, stress

## Hemispheric asymmetries in mental and neurodevelopmental disorders

While the vertebrate brain looks symmetrical at first sight, closer inspection reveals asymmetries on the functional and structural level (Güntürkün et al., 2020). In humans, functional asymmetries can be found in different cognitive systems like language production and perception (Häberling et al., 2016), spatial attention (Hattemer et al., 2011), and emotion processing (Gainotti, 2019). While the ontogenesis of asymmetries is still uncertain (Güntürkün and Ocklenburg, 2017), there is evidence that failure to establish typical asymmetries is associated with, for example, worse multitasking performance (Rogers et al., 2004). Moreover, several mental and developmental disorders show decreased hemispheric asymmetries (Berretz et al., 2020) which can manifest behaviorally as well as on the neurophysiological level. For instance, patients with schizophrenia display reduced lateralization in the planum temporale, a critical structure in language processing, which has been associated with the emergence of auditory verbal hallucinations (Oertel-Knöchel and Linden, 2011). For patients with major depressive disorder (MDD), there is evidence for reduced asymmetries in frontal alpha power in neurophysiological recordings through electroencephalography (EEG), which has been related to changes in emotion processing (Harmon-Jones, 2003). However, recent large-scale studies and meta-analyses in MDD patients did not find any associations with alterations in hemispheric asymmetries. For example, there is no link between altered handedness as a behavioral asymmetry and depression (Packheiser et al., 2021). Moreover, Kołodziej et al. (2021) concluded that changes in frontal alpha power asymmetries in depression are not sufficiently grounded on empirical evidence. Together with the recently reported absence of macrostructural brain asymmetry changes in depression (de Kovel et al., 2019a,b), this calls into question whether altered asymmetries play a role in the development and maintenance of depression at all.

## Heritability, etiology, and environment in disorders associated with altered asymmetries

The large heterogeneity between studies on atypical lateralization could be explained by differential incidences of reduced laterality within each disorder. We suggest that mental and developmental disorders express distinct subtypes of which only a few are associated with altered asymmetries. The prevalence of this subtype is associated with the ontogenesis of the individual disorder.

There are many possible symptom combinations displayed by patients with MDD (Park and Kim, 2021). At the core of the disorder, there are depressed mood and anhedonia. However, additional symptoms distinguish for example anxious or psychotic features (Malhi and Mann, 2018). This indicates that patients can have profoundly different experiences with a disorder while sharing the same diagnosis. With ~38% heritability (Kendler et al., 2006; Flint and Kendler, 2014), the role of genetics in the development of depression is notable but moderate. However, the influence of environmental factors like stressful life events in establishing MDD is irrefutable: severe illness, financial difficulties and unemployment, as well as interpersonal factors like separation and bereavement are major factor in the development of the disorder (Kessler, 1997; Kendler et al., 1999). Especially childhood maltreatment is strong risk factor for later depression (Li et al., 2016). Interestingly, different types of maltreatment, like physical or emotional abuse, are associated with different probabilities of developing depression (Infurna et al., 2016). This suggests that the type of environmental influence is associated with distinct disorder outcomes.

Compared to depression, schizophrenia and autism spectrum disorder (ASD) exhibit a much larger genetic component in their etiology expressed by their high rates of heritability. In the case of schizophrenia, heritability is estimated to lie between 73 and 78% (Hilker et al., 2018). On the non-genetic side, schizophrenia is associated with previous history of psychosis and parental socioeconomic status (Agerbo et al., 2015). However, as different cognitive phenotypes in schizophrenia show strong genetic influences, environmental and illness-related factors do not seem to decrease heritability (Blokland et al., 2017) even though they can influence epigenetic regulation (Richetto and Meyer, 2021). Although there is considerable heterogeneity in symptom expression among schizophrenia patients, two main subgroups have been characterized that differ in their disorganization, activation and psychosocial functioning (Picardi et al., 2012).

The heritability of ASD has been estimated to be ~80%, though there are differences depending on the country (Sandin et al., 2014; Colvert et al., 2015; Bai et al., 2019). Most environmental risk factors for ASD affect the intrauterine environment of offspring. To these count parental age, maternal intake of selective serotonin reuptake inhibitors and valproic acid, maternal diabetes and immune activation as well as intrauterine exposure to toxic chemical exposure (Colvert et al., 2015). While the heterogeneity in ASD is well accepted, recent research suggests that this heterogeneity is a result from contamination of data through lax diagnosis and not true biological differences (Mottron and Bzdok, 2020). Nonetheless, ASD remains a complex disorder with diverse etiologies and heterogeneous symptoms presentations, which should not be neglected in fundamental and clinical research (Hobson and Petty, 2021).

While heterogeneity within different disorders is typical, the diversity in etiology and symptom expression seems to be smaller for schizophrenia and ASD compared to depression.

## Higher number of endophenotypes in depression—A cause for a reduced association with altered asymmetries?

**Figure 1:**
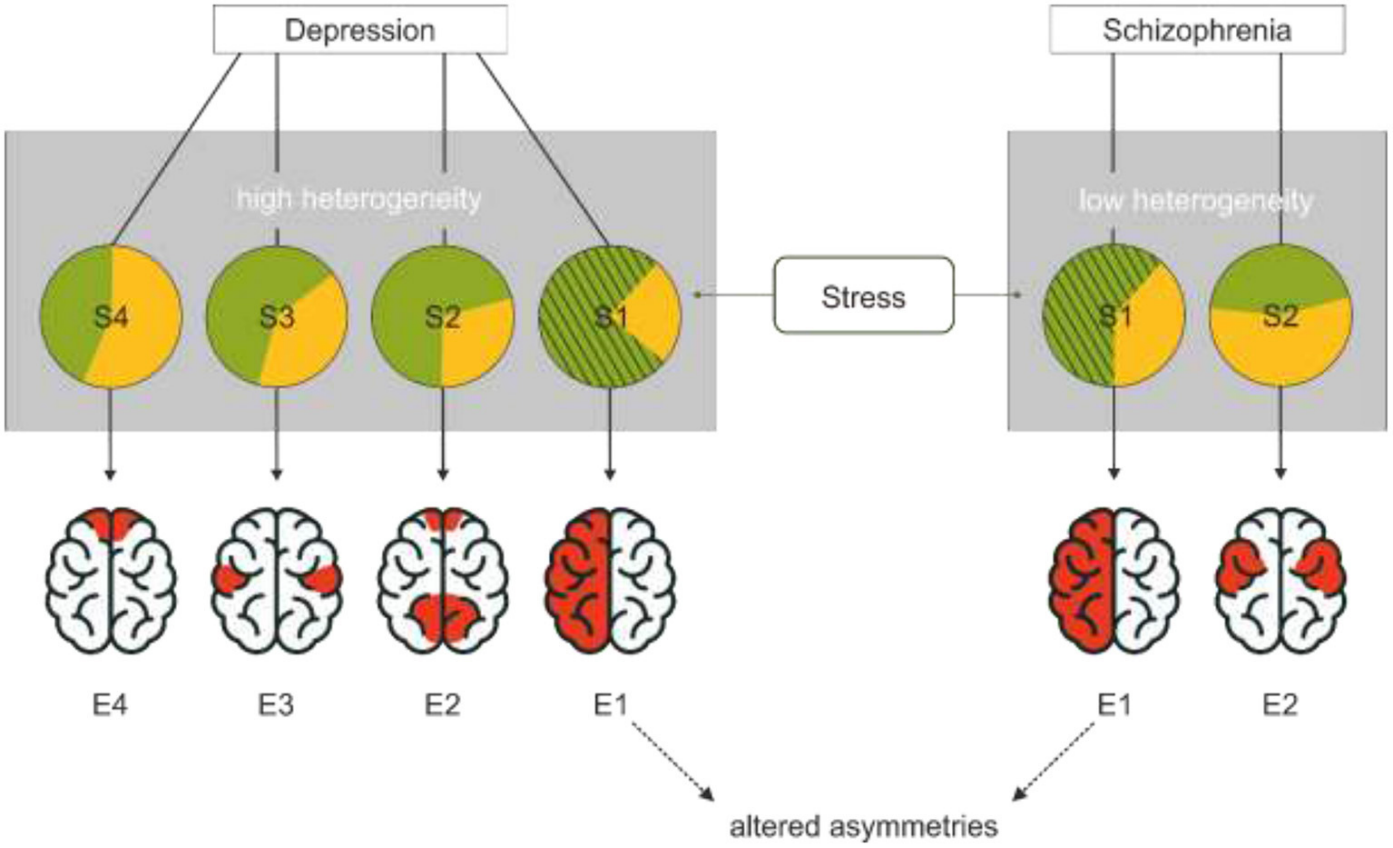
The association between variability of causes, endophenotypes and disorders. All these different symptom subtypes are subsumed under the same diagnosis (gray rectangles). Each circle represents a cluster of causes associated with different symptom clusters (S1–S4). Green indicates environmental influences while yellow indicates genetic influences. Different combinations of causes are possible for each disorder. A disorder with more distinct causation clusters forms more distinct symptom clusters. A disorder, in which only very specific clusters of causes lead to the clinical manifestation, fewer symptom clusters emerge. Different symptom clusters are associated with different endophenotypes. If only one phenotype is associated with altered asymmetry patterns, its probability of occurrence is lower for disorders with more distinct subtypes and thus risk factors. This model suggests that stress, shown as the hatched partial circle, is an environmental risk factor that specifically promotes the emergence of the endophenotype with altered asymmetries. This endophenotype refers to reduced typical asymmetries; in the case of depression, frontal alpha asymmetries would constitute a prime candidate while asymmetries in the language system are more central in schizophrenia.

While a unified theory of depression that seeks to integrate different symptoms and subtypes is very appealing (Dean and Keshavan, 2017), the underlying assumption that all types of depression are reasonably similar might obscure relevant neurological differences associated with these subtypes. In our model, we propose that different subtypes of symptom clusters expressed in depression might be associated with different neurostructural and — functional alterations and that the number and diversity of these subtypes is dependent on the diversity in ontogenesis of the disorder. Accordingly, a disorder with many possible combinations of causes might express more distinct endophenotypes than a disorder with few possible combinations of causes (Figure 1). If only one endophenotype of each disorder is associated with altered asymmetries, a disorder with a large variety of causes would have a lower proportion of patients with altered asymmetries than a disorder with few causes. Altered asymmetry in this case refers to reduced or inversed asymmetries that manifest in stronger bilateral hemispheric activation that can be driven by either increased activation of the typically sub-dominant hemisphere or decreased activation of the dominant hemisphere (Ocklenburg and Güntürkün, 2017).

For an example with arbitrary numbers, we would like to compare the ontogenesis of depression and schizophrenia. As genetics have a weaker impact on the development of depression compared to schizophrenia (Flint and Kendler, 2014; Hilker et al., 2018), the environment has a stronger influence in depression (Bagot et al., 2014) suggesting that more diverse environmental factors promote the development of depression than the development of schizophrenia. Thus, patients with the diagnosis of depression likely display higher heterogeneity in their etiology and symptoms than patients with schizophrenia. If depression would have four different causation profiles and schizophrenia has two different causation profiles each associated with a different endophenotype and one of these distinct endophenotypes for each disorder is associated with changes in asymmetries, there would be a proportionately higher rate of changes in asymmetry in schizophrenia (1/2) than in depression (1/4). In a large sample of patients, this would obscure the endophenotype of altered asymmetries in depression, while it could still be found in schizophrenia because the proportion of patients with this specific phenotype is higher.

## A link to intrauterine and early life stress and endophenotypes associated with altered asymmetries

We argue that certain disorders like depression, schizophrenia and autism display an endophenotype associated with altered asymmetries with the common environmental factor of stress (Berretz et al., 2020). In this context, intrauterine, early life and chronic stress could increase the probability of the emergence of disorders probably through unfavorable effects on brain development.

The strongest line of evidence on how stress affects the nervous system and the development of hemispheric asymmetries, in particular, comes from a series of rodent studies. In rats, lesions in the right mPFC have been associated with increased secretion of the stress hormone cortisol and stress-related ulcer formation after repeated stress exposure (Sullivan and Gratton, 1999). These hemispheric asymmetries have been related to the asymmetrical regulatory influence of the mesocortical dopamine system (Sullivan, 2004). This lateralized mesocortical inhibition has been shown to be altered in rats that were confronted with early life stress (Sullivan and Dufresne, 2006). Corroborating this finding, Mundorf et al. (2020) showed that acute stress only affected turning behavior in rats that had been exposed to early life stress. Similar findings could be observed in humans as prenatal stress leads to a reprogramming of the HPA-axis later in life (Lupien et al., 2009; O'Donnell and Meaney, 2017) and early life stress is associated with changes in behavioral asymmetries such as handedness (de Kovel et al., 2019b).

These stressors may promote the emergence of a subpopulation of patients that display alterations in asymmetries as has been shown in large samples in the UK Biobank (de Kovel et al., 2019a). It is feasible that these subgroups would display specific symptom combinations, like auditory verbal hallucinations in the case of schizophrenia that are distinct from symptom clusters expressed by patients without these stressors. Although our theory so far has not been empirically tested, individual studies provide preliminary evidence in its favor. For instance, it has been proposed that depression with childhood maltreatment, a critical traumatic and stressful early life event, constitutes a different subtype of the disorder than depression without childhood maltreatment (Teicher and Samson, 2013). This suggests that there are distinct subtypes of depression that result from differential environmental influences. Moreover, there is evidence that not all patients with depression display changes in frontal alpha asymmetries but that these changes constitute a distinct endophenotype of depression (Goldstein and Klein, 2014). Similarly, changes in language lateralization have been found to only be present in patients with schizophrenia who experience auditory verbal hallucinations (Ocklenburg et al., 2013). This suggests that not all patients with schizophrenia display changes in asymmetry but rather a subgroup that express a certain symptom subtype.

## Caveats of the theory

It has to be noted that this model is highly speculative and several of the claims made in this paper still need to be supported by more evidence. For example, it could be argued that schizophrenia may result from more genetic variants leading to more genetic combinations that result in schizophrenia than depression. While this is possible, these genetic variants and thus the resulting combinations leading to schizophrenia would be much rarer than the ones for depression since otherwise, both disorders should have similar prevalence. Similarly, it could be argued that the causes for depression might not be more eclectic but simply more common. However, in this case the outcome would be the same, namely a higher probability to develop depression due environmental factors than schizophrenia on the population level.

Our model does not necessarily assume that each endophenotype occurs with the same prevalence. It could be possible that the endophenotype of altered asymmetries occurs less frequently than any other phenotype. Moreover, this prevalence of this endophenotype may be different for different disorders.

An assumption that still needs to be supported is that the diversity of underlying causes relates to the diversity of symptom clusters and thus the diversity of associated endophenotypes. This assumption should be carefully considered as there is no direct evidence for it. If it does not withstand more thorough examination and experimental testing, this part of the model will clearly need to be updated in the future and we will be thankful for all future contributions. However, this would not be detrimental to the theory as a whole since the main goal was to propose that disorders have distinct endophenotypes and that only one endophenotype is associated with altered asymmetries. While we propose that the prevalence of this endophenotype is related to the diversity in causes, this is not essential.

## Outlook to the future

To test the core assumptions of our theory, a decently large sample of patients with, e.g., depression would be necessary. The patients would have to be exceptionally well phenotyped: not only typical covariates like age, education or time since onset or severity of the disorder would have to be collected. Rather, specific manifestations of symptom clusters as well as patient history of stress would be necessary. For the latter, traumatic childhood experiences, recurring or chronic stressors from other important developmental phases like adolescence and even maternal stressors would have to be accounted for. Additionally, different markers of asymmetry should be ascertained like macro- and microstructural asymmetries or frontal alpha asymmetries. Categorizing patients with and without asymmetry would then allow to look for possible differences between the two groups in displayed symptoms on the one hand and common etiological factors on the other hand. Although a study like this would be very elaborate and costly, insights gained from it would be worthwhile: not only could patients with different endophenotypes of the same disorder benefit differently from certain interventions; finding an association between stress and endophenotypes could inform neuroscience about the possible mechanisms of action mediating between the two variables (for example by changing asymmetry). Moreover, the usefulness of the data and the general idea behind the model does not end with asymmetries but could be extended to other biomarkers as well.

## Author contributions

GB conceptualized the article. GB and JP wrote the article. All authors contributed to the article and approved the submitted version.

## Funding

JP was supported by the German National Academy of Sciences Leopoldina (LPDS 2021-05).

## Conflict of interest

The authors declare that the research was conducted in the absence of any commercial or financial relationships that could be construed as a potential conflict of interest.

## Publisher's note

All claims expressed in this article are solely those of the authors and do not necessarily represent those of their affiliated organizations, or those of the publisher, the editors and the reviewers. Any product that may be evaluated in this article, or claim that may be made by its manufacturer, is not guaranteed or endorsed by the publisher.
